# A Concise Review of Extraction and Characterization of Chondroitin Sulphate from Fish and Fish Wastes for Pharmacological Application

**DOI:** 10.3390/cimb44090268

**Published:** 2022-08-28

**Authors:** Zannat Urbi, Nina Suhaity Azmi, Long Chiau Ming, Md. Sanower Hossain

**Affiliations:** 1 Department of Industrial Biotechnology, Faculty of Industrial Sciences and Technology, Universiti Malaysia Pahang, Kuantan 26300, Malaysia; 2 PAP Rashidah Sa’adatul Bolkiah Institute of Health Sciences, Universiti Brunei Darussalam, Gadong BE1410, Brunei; 3 Department of Biomedical Science, Kulliyyah of Allied Health Sciences, International Islamic University Malaysia, Kuantan 25200, Malaysia; 4 Faculty of Science, Sristy College of Tangail, Tangail 1900, Bangladesh

**Keywords:** FTIR, glycosaminoglycans, mass spectroscopy, NMR, purification

## Abstract

Chondroitin sulphate (CS) is one of the most predominant glycosaminoglycans (GAGs) available in the extracellular matrix of tissues. It has many health benefits, including relief from osteoarthritis, antiviral properties, tissue engineering applications, and use in skin care, which have increased its commercial demand in recent years. The quest for CS sources exponentially increased due to several shortcomings of porcine, bovine, and other animal sources. Fish and fish wastes (i.e., fins, scales, skeleton, bone, and cartilage) are suitable sources of CS as they are low cost, easy to handle, and readily available. However, the lack of a standard isolation and characterization technique makes CS production challenging, particularly concerning the yield of pure GAGs. Many studies imply that enzyme-based extraction is more effective than chemical extraction. Critical evaluation of the existing extraction, isolation, and characterization techniques is crucial for establishing an optimized protocol of CS production from fish sources. The current techniques depend on tissue hydrolysis, protein removal, and purification. Therefore, this study critically evaluated and discussed the extraction, isolation, and characterization methods of CS from fish or fish wastes. Biosynthesis and pharmacological applications of CS were also critically reviewed and discussed. Our assessment suggests that CS could be a potential drug candidate; however, clinical studies should be conducted to warrant its effectiveness.

## 1. Introduction

**Figure 1:**
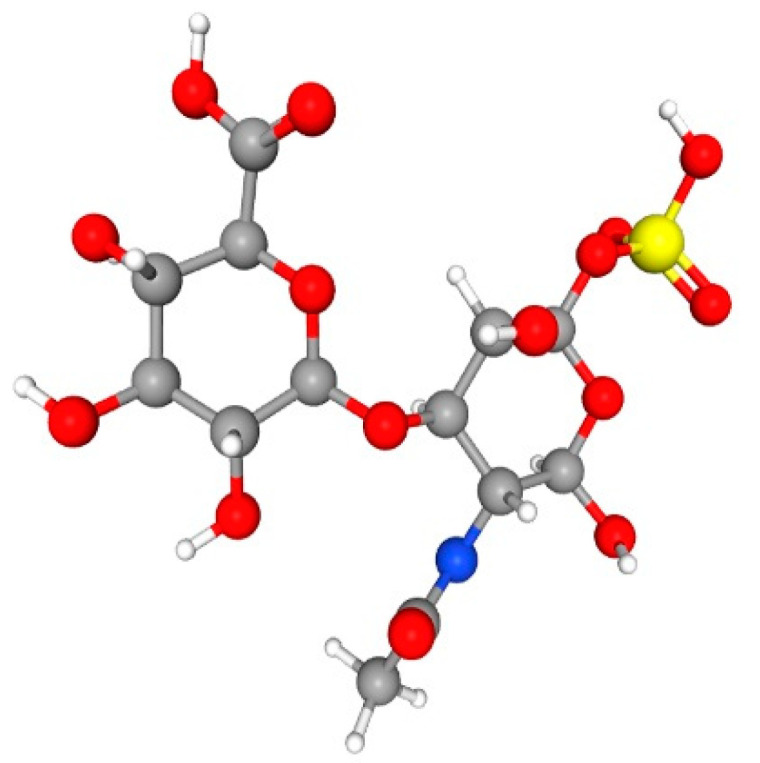
Chemical structure of Chondroitin sulphate. IUPAC name is (2*S*,3*S*,4*S*,5*R*,6*R*)-6-[(2*R*,3*R*,4*R*,5*R*,6*R*)-3-acetamido-2,5-dihydroxy-6-sulfooxyoxan-4-yl]oxy-3,4,5-trihydroxyoxane-2-carboxylic acid. The white stick shows a hydrogen bond, the ash ball shows carbon, the red ball shows O-linkage, the yellow ball shows the sulphate group, and the blue ball shows the amino group. This structure was retrieved from pubchem.ncbi.nlm.nih.gov (accessed on 2 November 2021).

Glycosaminoglycans (GAGs) are complex unbranched polysaccharides found throughout living organisms, including in internal compartments, on cell surfaces, and in the external environment [1,2,3,4,5]. Chondroitin sulphate (CS) is a sulphated glycosaminoglycan (GAG) found in cartilage and other body components that belong to the GAGs family (Figure 1) [6]. CS is a type of long linear polysaccharide made up of alternating N-acetylgalactosamine (GalNAc) and glucuronic acid (GlcA) saccharide units [7]. Sulphation of GalNAc residues at the 4-OH and/or 6-OH locations is possible, while GlcA may be sulphated at the 2-OH position [8]. CS is classified into four categories based on distinct sulphation patterns: CS-A = GlcA − GalNAc (4S), CS-C = GlcA − GalNAc (6S), CS-D = GlcA (2S) − GalNAc (6S), and CS-E = GlcA − GalNAc (6S) (4S, 6S) [9].

CS can be found on cell surfaces and in the extracellular matrix as proteoglycans. Cartilaginous tissues with elevated CS levels are distinguished by the presence of a thin collagen layer overlaid by an amorphous substance containing glycoproteins and proteoglycans. Currently, cartilaginous tissues from cows, pigs, and chickens are the most common sources of CS [10]. Pharmaceuticals, cosmetics, and food are just a few businesses that use CS. It is utilized to treat osteoarthritis [10], as regenerative medicine [11], and as a dietary supplement to prevent and treat articulation difficulties [12]. Commercial CS is currently limited due to the lack of a sustainable and risk-free source (viruses, prions, adulteration), which affects quality and production [13].

The global market value of CS was USD 1211.00 million in 2021. It is estimated that this value will increase by more than 41% by 2030 and reach USD 1709.00 million. The current demand for CS is being met mainly by the cartilage of sharks, cows, and pigs [14,15]. Alternative and safe ways to manufacture CS are now being researched [8]. Fish (both marine and freshwater) have been investigated as alternate halal sources of GAGs compared to terrestrial GAGs, including porcine [8,16,17]. Therefore, the CS production experiment has increased demand for its use as a versatile chemical in recent years. Fishbones, heads, gills, viscera, fins, eyeballs, and scales of marine and freshwater fish species are used to extract CS [17,18,19]. It has a different concentration and composition depending on where it comes from. CS-A is the primary constituent of CS derived from tracheal cartilage, whereas CS-C and CS-D are the main constituents of shark cartilage. Because the sulphation group can occur in various places, 16 distinct disaccharides can be produced. CS-B has sulphated N-acetylgalactosamine and glucuronic acid positions 4 and 2 [20].

**Figure 2:**
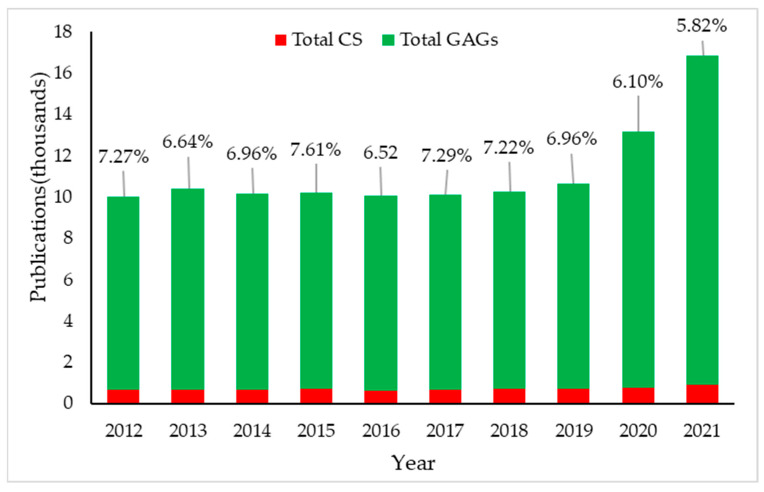
Annual publication statistics on scientific studies on Chondroitin sulphate (CS) from 2012 to 2021 in the Scopus database. The % value on the top of the bar graph shows the number of CS publications against the total glycosaminoglycans (GAGs) publications.

Even though the trend of study on CS has been static over the last decade, research on CS, particularly the role of CS in pharmaceutical research, has increased dramatically over the last two years due to high global demand and immense medical value. A systematic survey about the GAGs in the Scopus database using GAGs OR Heparin OR Heparan sulphate OR Chondroitin sulphate OR dermatan sulphate OR Keratan sulphate OR Hyaluronic acid within the *Title-Abstract-keywords* revealed a total of 316,975 papers, of which 244,469 research articles were found. Among this research, 104,732 papers were published from 2012 to 2021, of which 79,303 were research articles. Only CS was 7076 (6.76%) among the total papers on GAGs in the last 10 years, of which 5535 (6.98%) were research articles. Interestingly, during the last 10 years, researchers have actively focused on marine or fish species to isolate CS and evaluate pharmacological activities. Figure 2 show the yearly report of CS publications in comparison to GAGs in the Scopus database during 2012–2021. This data highlights how significantly CS is being studied for good science. Therefore, extending the understanding and insight into CS isolation, characterization, and health benefits by critically evaluating published literature is crucial and would be an excellent contribution to the scientific community.

Oral CS treatment has been shown to reduce osteoarthritic symptoms [21,22,23]. Pharmaceutical corporations are concerned about the use of anti-osteoarthritis medications. Clinical uses, unlike cosmetics or nutritional supplements, require highly pure CS. Researchers have determined the structure, physicochemical properties, and purity of each type of CS found in pharmaceuticals based on several analytical approaches. However, to the best of our knowledge, several procedures can be used to isolate CS/DS from other GAG species present in a mixture after tissue extraction, and it is also based on the researcher’s choice of which procedure they will follow. Therefore, this study critically reviewed and discussed the extraction, isolation, and characterization techniques to obtain CS from fish and fish wastes. Other sources of GAGs were also discussed where necessary to generalize the extraction, isolation, and characterization techniques.

## 2. Chondroitin Sulphate

### 2.1. Biosynthesis of CS

CS biosynthesis is a multistep and complex process achieved through enzymatic processes in the endoplasmic reticulum/Golgi compartments [24]. It starts with a GAG-protein linkage region covalently attached to specifically serine residues that are embedded in different core proteins [24,25,26]. There are multiple enzymes involved in this process (Table 1). The linkage regions are composed of a tetrasaccharide structure (GlcA1-3Gal1-3Gal1-4Xyl1), in which Gal and Xyl represent galactose and xylose residues, respectively, and are catalyzed by the tetrasaccharide glycosyltransferase. The cascade catalysis process of different enzymes (Table 1) finally forms CS (Figure 1). During polymerization, two GlcA C-5 epimerases convert GlcA residues in the Chondroitin skeleton to IdoA, transforming Chondroitin domains into dermatan domains [27,28]. Sulfotransferases using 3′-phosphoadenosine 5′-phosphosulfate as a donor substrate can site-specifically alter GalNAc or GlcA/IdoA residues [29]. Space–time-dependent expression and simultaneous action of these enzymes make the CS/DS chain structure complex, limiting structural and functional studies [24].

### 2.2. Fish and Fish Wastes for CS Production

Nowadays, substantial research is being conducted to isolate various biomolecules from fish samples for many uses, including pharmaceutical manufacturing. Several methods have been developed to recover CS from fish, including enzyme hydrolysis (ED), which was developed to recover a range of components from fish, including proteins and polysaccharides. Fishery wastes have been revealed to have the potential to be an excellent source for the extraction of valuable compounds.

In order to ensure that fish wastes are utilized to their best capacity, as shown in Table 2, CS was extracted from a variety of fish body parts [17,19,23,30,31,32,33,34,35]. These body parts are cartilage, the head, the eyes, the fins, and the fish’s skin. Several techniques were developed and optimized to separate the GAGs from the other polysaccharide complexes present in the tissues. To break down the structure and remove CS from the proteoglycans, different types of enzymes, solvents, and detergents can be used. Chemical hydrolysis of the tissue is typically conducted first to assure complete breakdown of the proteoglycan core, followed by the removal of proteins to recover specific GAGs from the resulting extracts, which is the most frequently used approach [36].

#### 2.2.1. Extraction and Isolation Technique

The first step of the purified CS production from fish samples is the extraction of crude GAGs either chemically or enzymatically. After separating the desired source sample of CS, delipidation using chloroform and acetone was conducted to remove fat adhering to the sample, followed by drying [33]. Proteolytic digestion using chemicals or enzymes is one of the most critical phases in this extraction process [37]. The removal of protein is aided by amyl alcohol and chloroform. However, the enzyme-free procedure is inefficient and reduces GAG yield [16]. The isolation of specific GAG from a particular tissue depends primarily on the optimum elimination of proteins by digestion with specific proteases [38].

Papain is the most often utilized enzyme, and it has been evaluated in several types of tissue samples for its ability to release GAGs [39]. Some other enzymes (i.e., alcalase, actinase E, trypsin) and chemicals (i.e., sodium chloride and cold acetone) were also used individually or in combination for GAGs preparation [35,38,40,41,42]. In enzymatic treatment for GAGs extraction, a combination of two enzymes, alkalase and flavourzyme, was evaluated [43]. This study revealed a higher GAG yield as well as a considerable reduction in treatment time. This might have occurred due to the synergistic effects of both enzymes. Therefore, using different combinations of enzymes is highly recommended for extracting a specific GAG yield. Design-expert software could be used to precisely and accurately design enzyme combinations applying factorial levels [44].

**Figure 3:**
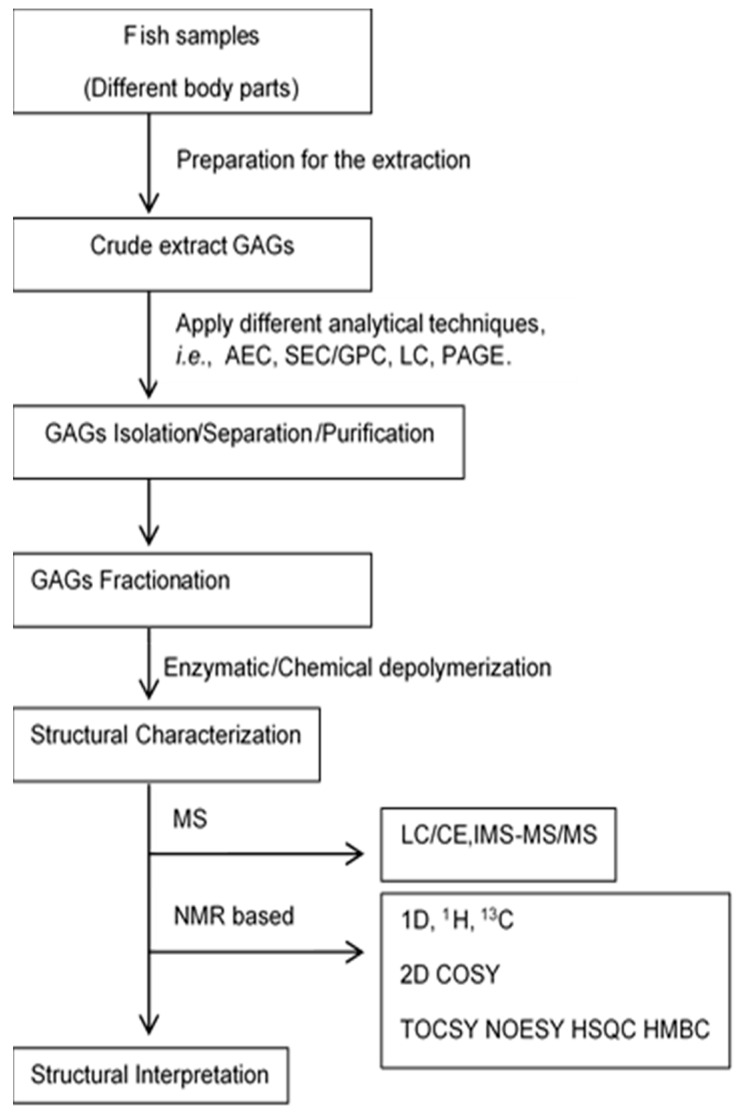
A common schematic presentation of different GAGs analysis. A specific enzyme (i.e., papain, alcalase, trypsin) or chemical solvent (i.e., chloroform, acetate, sodium acetate, chloroform-amyl alcohol) was used to produce CS.

The process for isolating chondroitin sulphate is dependent on the source material used; for example, cartilage from dogfish was successfully separated using a sodium acetate buffer solution and papain. An enzyme can be denatured at a higher temperature, and enzymatic digestion is frequently stopped if the enzyme is denatured at 100 °C for 15 min [46]. Activation of endogenous enzymes (autolysis) can effectively release GAGs from samples. Boiling water and digestion with a pancreatic enzyme can enhance the activation of endogenous enzymes. Approximately 70% of total CS was obtained from unmilled shark cartilage upon activation of endogenous enzymes. It is recommended that tissue degreasing with organic solvents and deproteinization with trichloroacetic acid be performed as key steps in extracting GAGs from tissues [36,44]. A general GAGs production process is shown in Figure 3.

#### 2.2.2. Purification and Characterization

Afterwards, the digestion (extraction/isolation) stage is followed by the purification or precipitation stage, which allows for the quantitative recovery of polysaccharides that have not been chemically degraded [47]. Fractionation of GAGs mixture is an important step for purifying a specific GAG such as CS. Many alcoholic solvents, including methanol, ethanol, and propanol, are used [44]. The use of alcohol during the subsequent phase of treatment is a critical step in the selective precipitation of CS from the hydrolysate and should not be overlooked. GAGs fractionated mixtures formed by repeated precipitation with methanol, ethanol, or propanol showed the same behavior [44]. Progressive precipitation with ethanol appears to be a comparatively successful approach for fractionating the GAGs mixtures, which may be considered a classical solvent intended to come into intimate contact with humans [21]. The fractionation of the GAGs was accomplished using isopropyl alcohol containing 2% sodium chloride [43,48]. Additionally, chromatographic methods were employed, but these are time-consuming and expensive [36,49,50].

Organic solvents used for the precipitation of GAGs have varying capabilities, and the precipitation is proportional to the amount and kind of solvent used. The precipitation efficiency of GAGs is influenced by other parameters, such as molecular mass, charge density, and structure of the solvents [51]. Organic solvents are preferable for the precipitation of sulphated GAGs such as CS, as they are stable [51].

Multiple techniques follow the precipitation step, including centrifugation, filtering, freezing, and lyophilization, to separate specific GAGs from the solvent (Figure 3) [44]. Afterwards, various analytical methods are used to purify GAGs to ensure high purity. Among the purification techniques available, ultrafiltration–diafiltration (UF–DF) is a frequently utilized approach that employs a size-based separation technique to eliminate impurities while simultaneously concentrating CS in solution [52,53,54]. Therapeutic biomolecules are purified using UF–DF in either a tangential or a crossflow mode. Membrane filters are used as filters in both ways. The biopharmaceutical industry relies on UF–DF to perform critical activities [55]. In the UF–DF purification process for the selective purification and protein permeation in the extraction process of CS, for example, a polyethersulfone membrane with a 30 kDa cut-off for the catshark (*Scyliorhinus canicula*) head, skeleton and fins [41] and a 30 and 100 kDa cut-off for the blue shark (*Prionace glauca*) head wastes [56] were used. Dialysis and chromatographic techniques are also used to purify isolated CS from impurities in the solution. Dialysis is not yet used for fish samples. It is used for CS purification from other animal sources to our best knowledge. For example, Li and Xiong [57] employed it as the final stage in purifying CS isolated from pig laryngeal cartilage. This technique was also used to purify CS isolated from buffalo cartilage [58]. Among the chromatographic methods, ion-exchange chromatography (IEC), fast protein liquid chromatography (FPLC) [59], low-pressure liquid chromatography (LPLC) [22], and high-performance gel permeation chromatography (HPGPC) [60] are commonly used. For the purification of CS, ion exchange resins, such as silica gel, were also used with traditional methods such as filtration [61]. The use of silica gel was found to improve the purity of the CS extraction process [62].

Through its interaction with functional proteins, CS controls a wide range of biological activities [63]. Therefore, structural and compositional analyses are the key to understanding its biological functions. As a highly versatile technique, mass spectrometry (MS) can be used to determine disaccharide composition, the molecular weight of larger oligosaccharides, the form of functional groups, and to a certain extent, the sequence of a specific GAG (i.e., CS). Conventional gel electrophoresis and blotting techniques can also acquire sequence-specific information by employing different reducing and nonreducing end labelling strategies [64]. However, this method requires a more extended analysis time and provides less informational content than MS. Liquid chromatography-mass spectrometry (LC-MS) methods, for example, can provide precise information about the structural diversities of GAGs discovered in tissue samples. By employing these approaches, researchers can acquire insight into the variations in the phenotypic distribution of GAGs in the tissue of diverse sources, developmental phases, and disease stages. Therefore, MS, especially when combined with LC (LC-MS), is arguably the most powerful method currently available for the structural analysis of GAGs.

Despite being the best tool for structural analysis and identifying positions of functional groups on GAGs, MS analysis has long been a formidable analytical challenge because of its high structural variability and proclivity for sulphate degradation. Undesirable sulphate loss is a big problem in MS analysis, and sulphate groups are quickly eliminated as neutral SO_3_ in the gas phase [65]. Protons catalyze the reaction; therefore, deprotonating sulphate groups or adduct formation with cations are effective options to prevent it [66]. Sulphate loss impairs mass spectrum interpretation and eliminates crucial sulphation number and position information. It is, therefore, critical to use delicate ionization procedures and source conditions to avoid activating fragile GAG ions in the gas phase [67].

To further extend the structural analysis of GAGs, nuclear magnetic resonance (NMR) can also be used to determine the structure of GAGs. GAG samples are prepared through rigorous separation processes and then subjected to MS and/or NMR analysis using top-down or bottom-up sequencing strategies, depending on the study performed [49]. For the rapid characterization of GAGs structure and composition, infrared (IR) and Raman spectroscopies could be complementary methods because these methods provide a complete “molecular fingerprint” of the studied sample. They are susceptible to the molecule’s structure, composition, and environment. These techniques are also non-destructive and require no external markers. With robust data analysis methods, spectroscopy can provide more insight into spectrum information and molecular-level phenomena. Combined with a microscope, they become susceptible procedures capable of probing at the micron level, requiring just tiny amounts of the sample [68].

The above discussion confirmed that CS structural analysis is a complex process. However, for quality and quantity assessment, having an accurate and reproducible analytical method is important. After extracting crude GAGs, enriching and purifying from impurities and chains of many sizes and isolating CS are crucial prior to successfully interrogating MS or NMR. Plenty of separation techniques have been used to purify and fractionate GAGs in order to isolate CS, namely AEC, size-exclusion chromatography (SEC), reverse phase liquid chromatography (RP-LC), reverse phase ion-pairing LC (RPIP-LC), and hydrophilic interaction chromatography (HILIC). Additionally, preparative polyacrylamide gel electrophoresis (PAGE) is also another good choice for CS purification. The purified CS from this technique can often be used directly to implement MS analysis for sequencing [49]. It is noted that Raman spectroscopy can be used for the quantitative identification of 4-sulfated and 6-sulfated isomeric CS. However, it can analyze both sulphated and non-sulphated polysaccharides, including heparin [69]. The most important features of this technique are that it is a non-destructive technique that requires a small amount of sample and is not time-consuming. In addition to Raman spectroscopy, the HPLC method can be used to separate and quantify underivatized CS/DS, unsaturated disaccharides (4- and 6-sulphated disaccharides) [70].

## 3. Prospective Pharmacological Application

The irregular expression of CS or GAGs causes many diseases, including tumors, viral infections, skeletal disorders, skeletal dysplasia, chondrodysplasia, multiple exostoses, Ehlers-Danlos syndrome, heart and kidney defects, immune deficiencies, glial scar (form after brain injury), and neurological abnormalities [71]. Therefore, it is expected that if CS expression can retain or reverse, it would affect the progress of respective diseases. Each disaccharide unit of CS contains only one sulphate group, which is a critical determinant of its pharmacological and pharmacokinetic activity [72]. Since the beginning of CS isolation, it has been mainly used for osteoarthritis treatment due to its anti-inflammatory properties [10]. Its uses in other interesting areas, including antiviral [73,74,75], anti-infections [76,77], regenerative tissue, and tissue engineering [11,78,79], are already well evaluated. In recent years, the role of CS in cancer cell development, neurodevelopment, and injury was established [80,81,82,83,84,85,86,87,88,89,90,91], indicating that CS could be used as a biomarker of cancer diagnosis and treatment by controlling its abnormal expressions.

The antiviral effects of CS were revealed in the fourth quartile of the last century. Transferring HIV from the mother to the postnatal breastfeeding child was severe concerning issue. Systematic experimentation revealed that human milk, particularly in the presence of CS, inhibits HIV envelope glycoprotein, gpl20, binding to its host cell receptor, CD4. This binding is the essential first step of HIV infectivity [75]. Designing nanomaterial and applying nanoformulation using CS could also be effectively used for antiviral infections for other viruses. The hydrogel produced by loading a hybrid of N,O-carboxymethyl chitosan (N,O-CMC) and oxidized CS showed excellent antibacterial properties due to the inherent antibacterial ability of N,O-CMC. Therefore, it can be used as a wound dressing material [92].

Anisha et al. [93] developed a chitosan–hyaluronan composite sponge incorporated with CS nanoparticles that showed enhanced swelling and blood clotting ability. This nanocomposite sponge showed more than 90% viability in cytocompatibility and cell adhesion tests using human dermal fibroblast (HDF) cells, which were carried out in two days. These findings indicated that CS nanoparticles containing nanocomposite sponges would be a potential candidate for wound dressing.

For more than two decades, a team of potential researchers demonstrated that CS proteoglycans (CSPGs) increased in the lesion and inhibited the growth of axons in a spinal cord injury (SCI) model [94]. Therefore, it inhibited the recovery of the function of the lesion. However, some other studies reported that injecting CSase ABC induces abnormal axon growth or enhances axon regeneration in zebrafish, adult rats, mice, and cats (see review [24]).

CSPGs play a key role in tumor growth and invasion due to the high expression of *CSPGs* in fast-growing tissues. Tumor cells are correlated with CS chains and their sulfation patterns. Moreover, CS can trigger a signaling pathway of tumor growth because negatively charged CS chains interact with many ligands and receptors [89]. However, many researchers recently established that remodeling the CS can be used as an anticancer agent [83,87,88].

A recent study demonstrated through in vitro and in vivo approaches that sturgeon-derived CS had the potential for treating colorectal cancer [87]. In vitro study showed inhibition of HCT-116 (human colon cancer cell line) proliferation in a dose-dependent manner of CS treatment, thereby enhancing excessive cell apoptosis. The further evaluation suggested that the inhibition might be associated with cell cycle arrest. Further extension of this study to xenograft HCT-116 in the mice model observed a significant inhibition of cell proliferation and apoptosis induction by CS treatment due mainly to the activation of b the *Bcl-2* family-associated mitochondrial pathway. Another study developed a three-dimensional porous chitosan-CS (C-CS) scaffold with 90–95% porosity and 143–166 μm pore size for evaluation against prostate cancer. This study showed a promising result where C-CS upregulated epithelial to mesenchymal transition marker expression, indicating physiological and pathological progress [95]. A prodrug nanoparticle system was developed using synthesized retinoic acid (RA)-conjugated CS (CS–RA) to target the Golgi apparatus that disrupts the structure of the Golgi apparatus and successfully inhibited the multiple metastasis-associated proteins expression in vitro and in vivo [83]. This study also demonstrated that CS-RA loading with paclitaxel inhibited migration, invasion, and angiogenesis in vitro and suppressed tumor growth and metastasis in 4T1-Luc bearing 6-week-old Balb/c female mice.

A recent study expounded a positive relationship between CS and colorectal cancer prevention. They reported that most of the studies included in their systematic review showed a positive relationship between the consumption of supplements containing CS and glucosamine and the prevention of colorectal cancer. However, weight gain was reported in those who consumed the supplement more frequently or simultaneously used non-steroidal anti-inflammatory drugs. However, this study did not confirm which drug was responsible for the weight gain [96]. Therefore, it is suggested that consuming CS more than the recommended dose should be avoided. Additionally, the therapeutic application of CS still confronts several challenges, such as immunogenicity, instability, and limited activity in vivo models. Many more preclinical studies and clinical studies with large population groups should be conducted before making any precise conclusion.

CS is classified as a slow-acting disease-modifying agent. It is widely used for the treatment of osteoarthritis; however, it shows its activity slowly [97,98,99]. It is capable of blocking degradative enzymes in vitro, such as leukocyte elastase and N-acetylglycosaminidase. This activity is only shown if the CS molecules are intact. However, unsulphated monomeric forms and breakdown products have not been tested or are yet unknown functions [100,101].

Many studies demonstrated that oral administration of CS improved human knee joint pains [102,103,104,105,106,107,108,109,110,111,112,113]. Even though glucosamine has been commercialized as a dietary supplement, oral CS bioavailability remains speculative, and conflicting results are found in the scientific literature. However, a recent clinical study reported positive results that the treatment of stage II osteoarthritis of the knee with Chondroguard^®^ is the most economically feasible in terms of cost-effectiveness. Additionally, CS activity entirely depends on the source of CS as its sulfation pattern varies, which is the key player in biological functions. Therefore, CS from different sources provides specific biological functions [105,114]. For example, fish cartilage shows different sulfation patterns compared to those of terrestrial vertebrates [115]. Therefore, CS from fish samples would be expected to show specific functions. Table 3 show some examples of pharmacological activities of CS isolated from fish.

## 4. Conclusions and Recommendations

Nowadays, fish waste production has increased significantly. CS production from fish wastes has an important role in producing biomacromolecules from waste materials useful for industrial applications. Additionally, it will comply with sustainable development goal 3: good health and well-being due to having plenty of medicinal benefits of CS. Much study has been conducted to ensure that CS is efficiently isolated in high yields and purity in various biomedical and pharmaceutical applications. CS are essential bioactive compounds used for many biological and pharmacological purposes. Furthermore, if CS is isolated from fish discards, it will add commercial value to the fishing industry as well as help in waste management.

Different fish organs are utilized to make CS, but the skin and cartilage are the most common source. Tissue hydrolysis, protein removal, and CS purification are all included. The amount of CS isolated by enzymes and/or solvents varies depending on the feedstock. The most common method is enzymatic digestion with papain, efficiently separating CS. LC (LC-MS) is arguably the best tool for the structural analysis of CS. However, IR and Raman spectroscopies could also be used as the fastest and most cost-effective methods for structural and compositional analysis of CS. Additionally, they are non-destructive, and no external marker is required.

The pharmacological functions of CS are promising, as discussed in the above sections. However, we explored that comparative pharmacological evaluation of CS isolated from different sources of fish and fish wastes is extremely limited. Clinical validation of CS isolated from different fish sources to assure safety and efficacy is also lacking. Therefore, elucidating the bioactivities specific to CS from different fish species, as well as other animal sources, is crucial to conducting more comprehensive studies.

## Figures and Tables

**Table 1:** Functions of enzymes involved in CS biosynthesis.

Stages	Enzyme Involved	Specific Function in CS Biosynthesis
Initiation	Glycosyltransferase	Catalyzed linkage region in the tetra-saccharide structure
Disaccharide unit formation	Xylosyltransferase	Catalyzed linkage between xylose and serine residue
	β1,4-galactosyltransferase I and β1,3-galactosyltransferase II	Catalyzed linkage between galactose and serine residues, in turn
	β1,3-glucuronyltransferase I	Catalyzed the formation of tetrasaccharide linkage region by adding GlcUA residue
	GalNAc transferase I	Catalyzed transfer of GalNAc residue to the nonreducing terminal GlcA residue and Chondroitin skeleton by adding GlcA and GalNAc residues in turn
Polymerization	GalNAc transferase II and GlcA transferase II	Form repeating disaccharide GlcA-GlaNAc in Chondroitin skeleton by alternative catalysis

Information extracted from Wang et al. [24].

**Table 2:** Extraction techniques and production of chondroitin sulphate from fish.

Source Name	Body Part	Enzyme	Extraction ^a^	Analytical Methods	Yield	CS	Other GAGs	Reference
Nile tilapia	Skin	Alcalase	Acetone, chloroform, methanol, TCA, NaCl, ethanol	AEC, NMR, AGE	0.15% DW	√	DS, HS	[33]
Pacu fish					0.18% DW			
Nile tilapia	Scale	Crude papain	Acetone, TCA, ethanol	IEC, AGE, NMR	0.86% DW	√	-	[30]
Nile tilapia	Bone residues (spine)	Papain	Ethanol, NaCl	TGA, DSC, FTIR, SEM	80% (residue: ethanol)	√	-	[45]
Nile tilapia	Skin	Papain	Sodium acetate, CPC, ethanol	IEC, AGE	10%	√	DS	[34]
Grey triggerfish	Skin	Alcalase	Sodium acetate, CPC, NaCl, ethanol	CAE	8.6%	√	DS, HS	[23]
Smooth dogfish					9.3%			
Monkfish Codfish Spiny Dogfish and Tuna	Bones	Papain (ED)	Acetone, Sodium acetate, NaCl, ethanol	AEC, AGE	Monkfish 0.34% Codfish 0.011% Dogfish 0.28%Tuna 0.023% (% *w*/*w* of bones)	√	-	[32]
Silver-banded whiting	Head	Lyase	Ethanol, NaCl, NaOH,	SEC, HPLC, NMR	70:20% and 50:30%,	√	HA	[35]
*Salmo salar* fish	Collagen-based scaffolds	Papain	Acetone, Sodium acetate, ethanol	Spectrophotometry	5%	√	-	[31]
Labeo rohita Piaractus brachypomus	Head	Papain	Acetone, TCA, ethanol, K-acetate	reverse-phase HPLC.		√	DS	[17]

^a^ These chemicals were used sequentially for the extraction of GAGs either for lipid or protein removal and precipitation of GAGs. For the details protocol, please see the respective cited paper. TCA: Trichloroacetic acid, AGE: Agarose gel electrophoresis, DW: Dry weight, CAE: Cellulose acetate electrophoresis, SEC: Size exclusion chromatography, K-acetate: Potassium acetate, CS: Chondroitin sulphate, DS: Dermatan sulphate, ED: Enzyme hydrolysis, HS: Heparan sulphate, HA: Hyaluronic acid, CPC: Cetylpyridinium chloride, IEC: Ion-exchange chromatography, SEM: Scanning electron microscope, AEC: Anion-exchange chromatography, TGA: Thermogravimetric analysis, DSC: Differential scanning calorimetry, HPLC: High-performance liquid chromatography, FTIR: Fourier transform infrared spectroscopy, GAGs: Glycosaminoglycans, √: Indicate the presence of CS.

**Table 3:** Pharmacological activities of CS from different fish species.

Fish Species	Char. of CS	Exp. Type (Model)	Dose and Admin.	Exp. Cond.	Pharmacology	Key Results	Ref.
Tilapia (*Oreochromis niloticus*) viscera	4-sulfated CS (59.6%); 6-sulfated CS (36.6%); Non-sulfated CS (3.4%)	In vitro (chemical analysis)	20, 40, 100, and 200 µg/mL	37 °C; 24 h	Antioxidant	↓ ROS (*p* < 0.01) highest level at 40 µg/mL	[116]
Nile tilapia (*Oreochromis niloticus*) viscera	CS-rich GAGs; Yield (0.15% of the freeze-dried sample)	In vitro (aPTT)	0.25 mg/mL	37 °C; 1 min	Anticoagulant	The Nile tilapia increased normal clotting time (2.3–2.8). The Pacu increased normal coagulation time (1.5–2.4)	[33]
Pacu (*Piaractus mesopotamicus*) viscera	CS-rich GAGs; Yield (0.158 of the dry sample)						
Grey triggerfish skins (GTCS)	Purity (99.2%); 41.72 kDa; 4-sulfated CS (59%); 6-sulfated CS (18.2%); Non-sulfated CS (3.5%)	In vitro (HCT116 cells)	10–200 µg/mL	1 × 10^7^ cells/mL; 37 °C; 24 h	Anticancer	↓ 70.6% for GTCS and 72.65% for SHCS (*p* < 0.05) at 200 µg/mL; No hemolysis; No cytotoxicity against the normal lymphocytes	[117]
Smooth hound skins (SHCS)	Purity (95.4%); 23.8 kDa; 4-sulfated CS (47%); 6-sulfated CS (14.6%); Non-sulfated CS (5.5%)						
Salmon cartilage	4-sulfated CS (30–40%); 6-sulfated CS (50–60%)	In vitro (Chemical analysis)	1, 2, 5, 10, 20 mg/mL	Chelating with divalent metal ions, Ca^2+^, Mg^2+^, Mn^2+^, or Zn^2+^	Antioxidant	Sulfated CS has significant antioxidant potency; CS chelation with Ca^2+^ or Mg^2+^ remarkably increased SOD radical scavenging; and CS chelation with Ca^2+^, Mg^2+^, Mn^2+^, or Zn^2+^ increased hydroxyl radical scavenging	[118]
Shark cartilage	4-sulfated CS (30%); 6-sulfated CS (40%)						
Ray cartilage	HMWCS; 6-sulfated CS (61.9%); 4-sulfated CS (27.0%); 2-sulfated CS-6-sulfated CS (8.5%); 142 kDa	In vitro (Hippocampal cells from E16 mice)	2 μg/well	2 × 10^4^ cells/cm^2^; 37 °C; 24 h	Neuritogenic activity	↑ Neurite outgrowth through the HGF signaling pathway; Specific binding of HGF to the CS	[119]
Skate (*Raja pulchra*)	HMWCS	In vitro and	5 or 50 mg/ml	37 °C; 10 or 30 min	Anti-obesity	Ø Pancreatic lipase activity; Ø Proliferation and lipid accumulation in mature adipocytes; HMWCS has greater lipase inhibitory activity than LMWCS;	[120]
		In vitro (mouse 3T3-L1)	Various concentrations and time points	37 °C; 15 min			
		In vivo (C57B/6 J mice; male; 4w)	50 mg/5 mL/kg/day; orally	8 w			
Skate cartilage	CSE, CS	In vivo (Mice)	200, 400 mg/kg; orally	3 consecutive days	Antiinflammation, hepatic dyslipidemia	↑ Hepatic antioxidant enzyme expression levels; Ø Inflammatory factors; ↓ Serum lipid; ↓ hepatic sterol regulatory element-binding proteins expression; ↓ MAPK; and ↓ Apoptopic factors	[121]
Sturgeon cartilage	AMWCS; 4-sulfated CS (88.8%); 8 kDa	In vitro (Fibroblast)	100 µg/ml	1 × 10^3^ cells/well; 37 °C; 24 h	Wound healing	↑ cell adhesion; ↑ Proliferation and migration on fibroblasts; and ↑ MAPK signaling pathways	[122]
Sturgeon backbone	AMWCS; 6-sulfated CS (60%); 43 kDa						
Sturgeon skull	AMWCS; non-sulfated CS (74.2%); 38.5 kDa	In vitro (Rabbit blood)	1, 3, 5 mg/ml	360 µL of platelet-poor plasma; 15 min	Anticoagulant, anti-platelet, and thrombolysis	↑ aPTT; ↑ TT; Ø Platelet aggregation; Dissolved platelet plasma clots; Sturgeon backbone CS was stronger than sturgeon skull CS	[123]
Sturgeon backbone	AMWCS; 4-sulfated CS (37.8%); 6-sulfated CS (59.6%); 49.2 kDa						
Shark cartilage	LMWCS (75.7%); 3.9 kDa	In vitro (PC12; SH-SY5Y)	50, 100, 200 µg/ml	0.5 × 10^4^ cells/well; 24 h	Neuroprotection	× Cell viability loss and apoptosis; ↓ Intracellular Ca; ↓ ROS levels; ↓ MMP depolarization; and ↓ Protein expression of Caspase-3	[124]
Shark cartilage	LMWCS (75.7%); 3.9 kDa	In vivo (Male Balb/c mice; 8 w)	50, 150, 450 mg/kg; perorally (p.o.)	Daily; 31 days	Neuroprotection	Improved the cognitive impairment; ↑ ChAT level; ↑ SOD; and ↑ GSH-Px; ↓ MDA level; and ↓ AChE level; ↓ Pyramidal cells of CA1 regions; Ø Protein expression of Bax/Bcl-2 and Caspase3, -9.	[124]
Small sea fish	CS Extracts (CP)	In vitro (CHON-001)	CP 0.2%, 0.3% (*v*/*v* in culture medium); CS 3, 200 µg/mL	48, 72 h	Osteoarthritis	× Chondrocytes decline; Ø Osteo-articular inflammation Ø Apoptosis; and ↑ Proliferation rate	[125]

Admin.: Administration, Chac.: Characteristics, Cond.: Conditions, Exp.: Experimental, LMWCS: Low molecular weight chondroitin sulphate, ROS: reactive oxygen species, MMP: mitochondrial membrane potential, ×: Block, Ø: Suppress/Inhibit, ↓: Decrease, ↑: Increase, ChAT: Choline Acetyltransferase, SOD: Superoxide dismutase, GSH-Px: Glutathione peroxidase, MDA: Malondialdehyde, AchE: Acetylcholinesterase, Ca: Calcium, CHON-001: human chondrocytes cell line, CSE: Chondroitin sulphate-rich extracts, MAPK: mitogen-activated protein kinase, AMWCS: Average molecular weight Chondroitin sulphate, aPTT: Activated partial thromboplastin time; TT: Thrombin time, HGF: Hepatocyte growth factor, HCT116: Human colon carcinoma.

## Data Availability

Not applicable.
